# Genomic insights into the versatility of the plant growth-promoting bacterium *Azospirillum amazonense*

**DOI:** 10.1186/1471-2164-12-409

**Published:** 2011-08-12

**Authors:** Fernando H Sant'Anna, Luiz GP Almeida, Ricardo Cecagno, Luciano A Reolon, Franciele M Siqueira, Maicon RS Machado, Ana TR Vasconcelos, Irene S Schrank

**Affiliations:** 1 Centro de Biotecnologia, Universidade Federal do Rio Grande do Sul, Av. Bento Gonçalves, 9500, Campus do Vale, Porto Alegre, RS, Brazil; 2 Laboratório Nacional de Computação Científica (LNCC), Petrópolis, RJ, Brazil; 3 Departamento de Biologia Molecular e Biotecnologia - Centro de Biotecnologia, Universidade Federal do Rio Grande do Sul, Av. Bento Gonçalves, 9500, Campus do Vale, RS, Brazil

## Abstract

**Background:**

The species *Azospirillum amazonense *belongs to a well-known genus of plant growth-promoting bacteria. This bacterium is found in association with several crops of economic importance; however, there is a lack of information on its physiology. In this work, we present a comprehensive analysis of the genomic features of this species.

**Results:**

Genes of *A. amazonense *related to nitrogen/carbon metabolism, energy production, phytohormone production, transport, quorum sensing, antibiotic resistance, chemotaxis/motility and bacteriophytochrome biosynthesis were identified. Noteworthy genes were the nitrogen fixation genes and the nitrilase gene, which could be directly implicated in plant growth promotion, and the carbon fixation genes, which had previously been poorly investigated in this genus. One important finding was that some *A. amazonense *genes, like the nitrogenase genes and RubisCO genes, were closer phylogenetically to Rhizobiales members than to species of its own order.

**Conclusion:**

The species *A. amazonense *presents a versatile repertoire of genes crucial for its plant-associated lifestyle.

## Background

The genus *Azospirillum *(α-proteobacteria class) encompasses free-living bacteria that can improve the growth of many economically important plants, mainly cereals (for an extensive review, see [1]). Therefore, these microorganisms are considered as plant growth-promoting rhizobacteria (PGPR). Species of this genus are widely distributed in nature, living in soils of tropical, subtropical and temperate regions all over the world. Several aspects of their physiology seem to be related to a plant stimulatory effect, notably their ability to synthesize phytohormones. Although these microorganisms are able to fix atmospheric nitrogen, the exact contribution of this process to plant growth is still disputable [1-3].

So far, fifteen species of the *Azospirillum *genus have been described ([4] and references therein). However, most research efforts have been dedicated to the species *Azospirillum brasilense*, neglecting the potential offered by the biological diversity of this genus.

The bacterium *A. amazonense*, the focus species of this study, was initially isolated from forage grasses grown in the Amazon region. Further studies revealed its broad ecological distribution, as it is also found in association with the roots of gramineous plants like rice, maize, sugarcane and sorghum [5,6]. This species is phylogenetically closer to *Azospirillum irakense *and *Rhodospirillum centenum *(also known as *Rhodocista centenaria*) than to *A. brasilense*. Unlike the latter, *A. amazonense *can use sucrose as a sole carbon source and is better adapted to acid environments [6].

In order to access the valuable information that genomic sequences can provide on the physiology of azospirilla, there have been independent efforts by research groups in sequencing their genomes. Currently, the genomes of three members of the *Azospirillum-R. centenum *group are available: *Azospirillum *sp. B510 [7], *A. brasilense *Sp245 [8] and *R. centenum *SW [9]. Although many years have passed since the discovery of *A. amazonense*, there is scarce information about this species. Nevertheless, a recent study under greenhouse conditions showed that especially *A. amazonense *Y2 (wild-type strain) contributed to the growth of rice plants by means of biological nitrogen fixation [10], showing its potential for use as an agricultural inoculant. Therefore, the objective of our study was to sequence the *A. amazonense *Y2 genome and to analyze specific regions that could exert fundamental roles in its survival in the soil and in its ability to promote plant growth.

## Results and Discussion

### General features of the A. amazonense draft genome database

The *A. amazonense *Y2 presents four replicons with the following estimated sizes: 2.7 Mbp, 2.2 Mbp, 1.7 Mbp and 0.75 Mbp [11] The genomic G+C content of *A. amazonense *Y2 is 66.89%. The draft genome sequence consists of 7.044.835 bp divided in 1617 contigs. The average gene length is 1080. Currently, there are 3319 predicted CDS, where 2299 have sequence similarity to known genes, 501 are homologs to genes of unknown function and 519 are hypothetical genes exclusive to *A. amazonense*.

### Taxonomic features of A. amazonense

**Figure 1:**
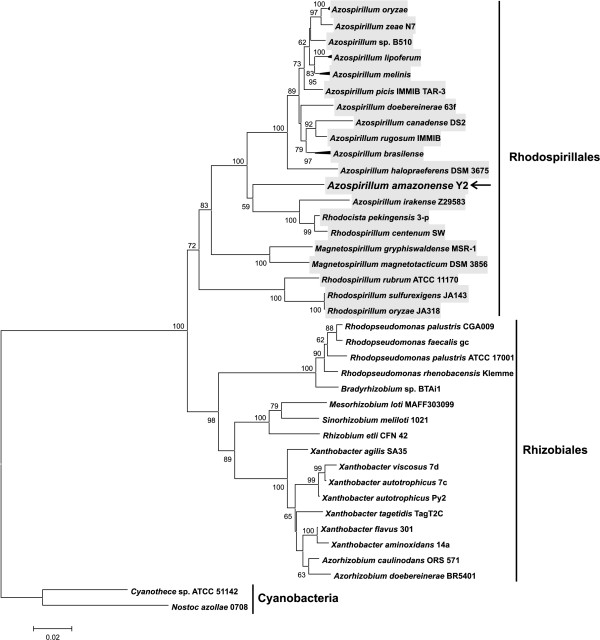
**The evolutionary history of 16S rDNA sequences from members of the orders Rhodospirillales and Rhizobiales**. The tree was constructed using the Neighbor-Joining method with 1000 bootstrap replicates (percentages greater than 50% are shown next to the branches). The tree is drawn to scale, with branch lengths in the same units as those of the evolutionary distances used to infer the phylogenetic tree. There were a total of 1254 positions in the final dataset. The species *Azospirillum amazonense *is indicated by an arrow. The gray boxes indicate members of the order Rhodospirillales.

In this study, a phylogenetic tree was constructed using the 16S rDNA sequences from microorganisms belonging to the orders Rhodospirillales and Rhizobiales (Figure 1). The resulting phylogenetic tree clearly shows a split between these orders. The outermost clade containing all the *Azospirillum *species divides in two main subclades: one containing *A. amazonense, A. irakense, Rhodocista pekingensis *and *R. centenum*, and another containing the other *Azospirillum *species. This result is in agreement with previous studies, showing the close evolutionary relationship between *A. amazonense *and *R. centenum *[12-14], and is also supported by the greater number of *A. amazonense *genes (22%) with best BLAST hits (KEGG Database) to *R. centenum *genes.

Another relevant feature of the 16S rDNA phylogenetic reconstruction is that *R. centenum *does not cluster with other *Rhodospirillum *species, suggesting that the genus nomenclature of *R. centenum *is not appropriate, as has been pointed out by previous publications [13,15].

### Carbon metabolism

Azospirilla display versatile carbon metabolism in order to support their lives in the soil. *A. amazonense *is capable of growing on various disaccharides, hexoses and pentoses and a previous study suggested that *A. amazonense *is able to catabolize carbohydrates exclusively through the Entner-Doudoroff pathway (ED pathway) [16]. The genes encoding the key enzymes of this pathway, 6-phosphogluconate dehydratase and 2-dehydro-3-deoxy-phosphogluconate aldolase (KDPG aldolase) (Additional file 1), were found in the *A. amazonense *genome and seem to be organized as an operon. This same study also suggested that the glycolysis pathway (Embden-Meyerhof-Parnas pathway) was inoperative in *A. amazonense*, because no activity of 6-phosphofructokinase and fructose bisphosphate aldolase was detected in crude extracts [16]. However, predicted genes encoding those enzymes were found in the *A. amazonense *genome (Additional file 1). Therefore, although the genomic approach indicates that most probably *A. amazonense *is also able to consume carbohydrates via glycolysis, this catabolic feature should be experimentally re-tested.

As stated previously, one of the main differences between *A. amazonense *and *A. brasilense *is that *A. amazonense *is capable of consuming sucrose as the sole carbon source [6]. In the genome of *A. amazonense*, a predicted gene that codes for a putative α-glucosidase was identified (Additional file 1). This enzyme converts sucrose to glucose and fructose, substrates that can be promptly consumed by catabolic pathways.

The *A. amazonense *genome also harbors homologs of the genes *salB *and *salA *of *A. irakense *(Additional file 1). These genes encode β-glucosidases, enzymes implicated in the acquisition of glucose by means of the hydrolysis of aryl-β-glucosides, such as salicin [17].

Bacteria of the genus *Azospirillum *produce high levels of poly-β-hydroxybutyrate (PHB), the energy and carbon storage source utilized under nutritional stress conditions [2,18]. The essential genes for PHB biosynthesis are present in the *A. amazonense *genome: *phbA *(β-ketothiolase), *phbB *(aceto acetyl coenzyme A reductase) and *phbC *(PHB synthase) (Additional file 1). Furthermore, the *phaZ *gene that encodes a PHB depolymerase (Additional file 1), the first enzyme of the PHB degradation pathway, was also found in its genome.

**Figure 2:**
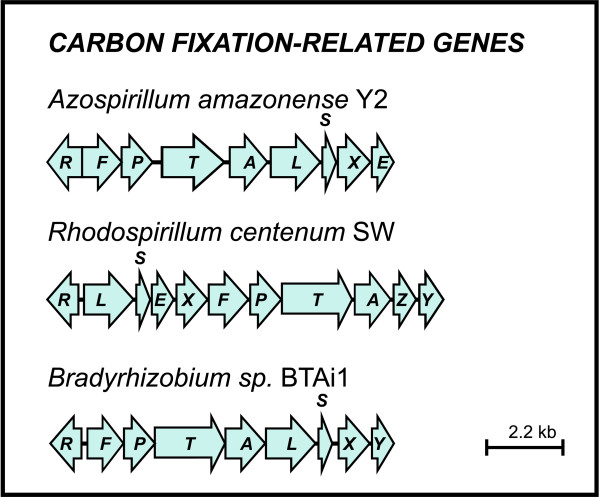
**Organization of the carbon fixation gene cluster across different species**. Arrows represent genes and their respective direction of transcription.

**Figure 3:**
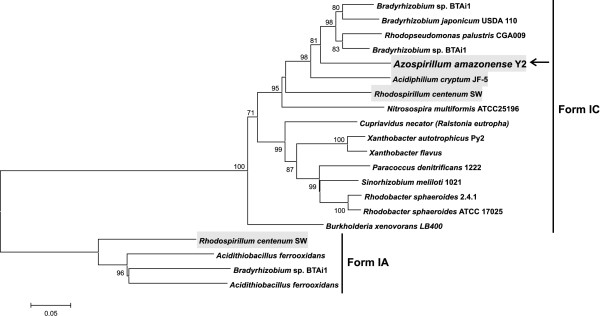
**Phylogenetic tree based on the concatenated CbbL-CbbS RubisCO amino acid sequences**. There were a total of 557 positions in the final dataset. Details are as shown in Figure 1.

One of the most surprising features of the *A. amazonense *genome is the presence of a gene cluster implicated in carbon fixation (the Calvin-Benson-Basham cycle) (Figure 2 and Additional file 1). The main genes of this cluster are the genes *cbbL *and *cbbS*, and they encode, respectively, the large and small subunits of ribulose-1,5-bisphosphate carboxylase (RubisCO). This enzyme is responsible for the incorporation of carbon dioxide in a molecule of ribulose-1,5-bisphosphate, generating two molecules of 3-phosphoglycerate, which can subsequently be used in biosynthetic pathways. A phylogenetic analysis of the concatenated RubisCO small and large subunits of *A. amazonense *revealed that they belong to the Form IC of RubisCOs (Figure 3). This type of enzyme is commonly found in α-Proteobacteria and it is adapted to environments with medium to high CO_2 _and the presence of O_2 _(in general, RubisCOs also have affinity to O_2 _and high levels of this molecule can inhibit CO_2 _fixation) [19]. So far, there have been no reports showing that *A. amazonense *has autotrophic behavior. However, from the *Azospirillum *group, at least *R. centenum *and *A. lipoferum *are known to be capable of growing autotrophically by means of RubisCO [9,20], unlike *Azospirillum *sp. B510 and *A. brasilense *Sp245, which do not contain Form I or II of RubisCOs ("true" RubisCOs) encoded in their genomes.

The RubisCO phylogenetic reconstruction also indicated the close relationship of the *A. amazonense *enzyme with those from members of the family Bradyrhizobiaceae (order Rhizobiales) (Figure 3), namely *Rhodopseudomonas palustris *and *Bradyrhizobium *spp. In fact, the genetic organization of the carbon-fixation cluster of *A. amazonense *resembles that found in *Bradyrhizobium *sp. BTAi1, contrasting with the genetic organization of *R. centenum*. These incongruities, i.e. the genetic organization and phylogenetic relationship closer to Bradyrhizobiaceae members than to *R. centenum*, suggests that horizontal gene transfer may be an important driving force in the evolution and dispersion of RubisCOs in Proteobacteria.

### Nitrogen metabolism

*Azospirillum *species are able to utilize distinct nitrogen sources, including ammonia, nitrate, nitrite, dinitrogen and amino acids [3,21]. The *A. amazonense *species has several genes implicated in nitrogen metabolism, which encode transporters, enzymes and regulatory proteins (Additional file 1).

Ammonia is the central compound of nitrogen metabolism and the preferred nitrogen source of many microorganisms. In general, nitrogen sources other than ammonia are converted into it to be assimilated [22]. The *A. amazonense *genome contains genes that are implicated in this conversion of alternative nitrogen sources, like nitrate/nitrite, urea and dinitrogen (Additional file 1). Once available, ammonia can be incorporated into the metabolism by the glutamine synthetase (GS)/glutamine:oxoglutarate aminotransferase (GOGAT) pathway, the genes for which are also encoded in the *A. amazonense *genome (Additional file 1).

Overall, the conversion of nitrogen compounds to ammonia expends some energy and, therefore, the metabolic pathways implicated in this process are strictly regulated to minimize energy waste. The central regulators of nitrogen metabolism are the PII proteins [22,23]. Three PII homolog genes (*glnB, glnK *and *glnK2*) were found in the *A. amazonense *genome (Additional file 1). The *glnK *gene and the *glnB *gene have ortholog counterparts in *Azospirillum *sp. B510, *A. brasilense *Sp245 and *R. centenum*. The *glnK *gene is upstream of the *aat *gene (aminotransferase) and the *glnB *is upstream of the *glnA *gene (glutamine synthetase) [24]. The third gene, *glnK2*, which is located downstream of the *amtB *gene, is absent in *A. brasilense *Sp245, *Azospirillum *sp. B510 and *R. centenum*, although this genetic association is frequently found in diverse prokaryotes [25].

The PII protein interactions with transporters, transcription factors and regulatory enzymes are well-established in the literature (for a review, see [22,23]) and the potential interaction targets found in the *A. amazonense *genome will be briefly discussed. One putative target is the *glnD *gene that codes for an uridilyl-transferase, an enzyme that uridylylates the PII proteins under nitrogen-limiting conditions [23]. Other potential targets found in the *A. amazonense *genome are two ammonium transporters, encoded by the *amtB *genes, which in the presence of high nitrogen levels are inhibited by PII proteins [26]. The adenylyltransferase enzyme (encoded by the *glnE *gene, Additional file 1) which regulates glutamine synthetase via covalent modifications [27] could also interact with PII proteins [28].

The analysis of the *A. amazonense *genome revealed the presence of the NtrBC and NifA systems (Additional file 1), which are PII-regulated signal transduction systems responsible for the coordination of genes implicated in the scavenging of alternative nitrogen sources [23]. Both NtrC and NifA rely on the presence of the sigma N factor (also known as RpoN, or sigma 54) to activate the transcription of specific genes [29], which is also present in the *A. amazonense *genome (Additional file 1).

One of the main characteristics of the *Azospirillum *species is that they are able to fix nitrogen, i.e. convert N_2 _to ammonia, by means of the nitrogenase enzyme complex. The main genes implicated in this process are known as *nif *genes, and they are highly conserved among nitrogen-fixing proteobacteria [30].

**Figure 4:**
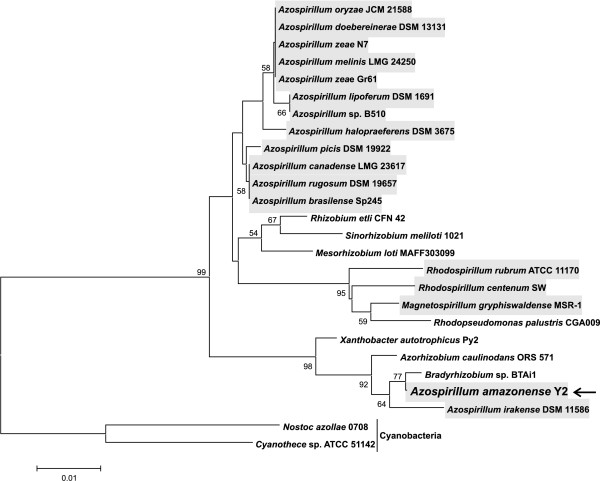
**Phylogenetic tree based on the partial *nifH *gene from members of the orders Rhizobiales and Rhodospirillales**. There were a total of 215 positions in the final dataset. Details are as shown in Figure 1.

A preliminary BLAST analysis showed that the *A. amazonense nif *genes exhibit high similarity with genes of some species of the order Rhizobiales (Additional file 1). Since these observations were unexpected, the phylogenetic history of *nifH *was reconstructed utilizing sequences from species of the orders Rhodospirillales and Rhizobiales. The resulting *nifH *tree (Figure 4) was clearly incongruent to the 16S rDNA tree (Figure 1): in the *nifH *tree, *A. amazonense *and *A. irakense *grouped with *Bradyrhizobium *sp. BTAi1, *Xanthobacter diazotrophicus *and *Azorhizobium caulinodans*, instead of grouping with other *Azospirillum *species.

**Figure 5:**
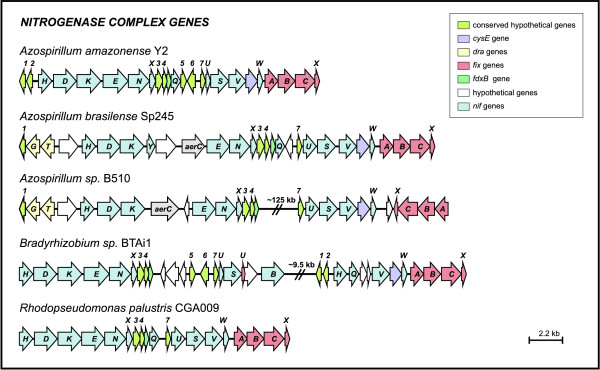
**Organization of the nitrogen fixation gene cluster across different species**. Arrows represent genes and their respective direction of transcription. Genes are colored as depicted in the upper box.

The genetic organization of the *nif *genes between the Rhodospirillales and Rhizobiales bacteria is somewhat homogeneous (Figure 5). As demonstrated in Figure 5, some features of the *nif *cluster of *A. amazonense *are exclusively similar to the homolog cluster of the species *Bradyrhizobium *sp. BTAi1, such as the presence of three conserved hypothetical genes that are indicated by the numbers 2, 5 and 6. On the other hand, *Azospirillum *sp. B510 and *A. brasilense *have the *aerC *gene between the *nifHDK *and *nifENX *operons and the *draG *and *draT *genes in the upstream region of the *nifH *gene, features not shared with the *nif *cluster of *A. amazonense*. The genes *draG *and *draT *code for a post-translational control system of the nitrogenase, which are not present in *A. amazonense *and *R. centenum *[9,31].

The domain composition of the deduced NifE and NifU proteins is also evidence that the nitrogenase complex of *A. amazonense *is more distantly related to *Azospirillum *spp. (with the exception of *A. irakense*) than to some Rhizobiales species (i.e. those from the genus *Bradyrhizobium, Xanthobacter *and *Azorhizobium*). The NifE protein of *A. amazonense *(like the *Bradyrhizobium, Xanthobacter *and *Azorhizobium *proteins NifE) has a bacterioferritin-associated ferredoxin [2Fe-2S] binding domain (BFD), not present in the NifE protein from *A. brasilense *Sp245 and *Azospirillum *sp. B510, and *R. palustris *(Rhizobiales) (Additional file 2). Furthermore, its NifU protein is smaller than those of *A. brasilense *Sp245, *Azospirillum *sp. B510 and *R. palustris *CGA009 because it does not contain the BFD and an N-terminal NifU domain, which are present in the NifU protein of the latter species (Additional file 2).

Therefore, taken together, these results indicate that complex events occurred in the evolution of the nitrogenase system in these bacteria, for instance, horizontal gene transfers and/or gene duplication followed by differential gene loss, culminating in the current distribution of the *nif *genes among the members of these taxonomic orders.

Nitrogen fixation is a very energy-demanding process and it is not surprising that the nitrogenase system is elaborately regulated. In all diazotrophic species of the Proteobacteria examined so far, the transcriptional activator NifA and the sigma N alternative RNA polymerase-associated factor are the master regulators of nitrogen fixation genes [32]. Sequence motifs similar to the consensus region of sigma N and NifA binding sites are present upstream of the *nifH *gene, the homolog of "*orf2*" (indicated by the number 7 in Figure 5) of the *orf2nifUSVorf4 *cluster from *A. brasilense *[33] and the *nifB *gene (Additional file 3).

### Energy production and conversion for nitrogen fixation

Nitrogen fixation demands the systematic action of different genes. The *fix *genes are essential for this process, and they encompass different functional categories. These genes were found in the *A. amazonense *genome divided into three main clusters, namely *fixABCX, fixLJ-fixK *and *fixNOQP-fixGHIS*.

The *fixABCX *genes from *A. amazonense*, responsible for electron transfer to nitrogenase, are located downstream of the *nifW *gene (Figure 5). As seen in Figure 5, this genetic cluster is highly conserved among the analyzed species, and it is tightly associated with the *nif *cluster. Putative NifA and sigma N binding sites were found upstream of the operon *fixABCX *from *A. amazonense *(Additional file 3), indicating that these transcription factors could be key elements for the expression of this operon. This evidence is corroborated by reports showing that the operon *fixABCX *is regulated by the NifA protein in *A. brasilense *and *Rhizobium *spp. [34-36].

The *A. amazonense *genome also possesses a gene cluster including *fixLJ *and *fixK*. In symbiotic diazotrophs, the transcription of *fix *genes involves the oxygen-responsive FixLJ two-component system. The FixL protein, in the absence of oxygen, autophosphorylates and transfers the phosphate group to FixJ. Finally, the phosphorylated FixJ activates the expression of FixK, which activates the transcription of genes required for microaerobic growth [32].

Molecular nitrogen reduction requires high levels of energy under microaerobic conditions. The *fixNOQP *and *fixGHIS *genes encode membrane-bound cytochrome c oxidase and the redox process-coupled cation pump, respectively, which are intimately implicated in respiration under microaerobic conditions, supplying energy for nitrogen fixation [37,38]. These clusters were found *in tandem *in the *A. amazonense *genome (Additional file 1), and they show identical organization in many diazotrophic α-Proteobacteria, like *R. centenum, A. brasilense *Sp245, *R. palustris *and *Bradyrhizobium *spp.

**Figure 6:**
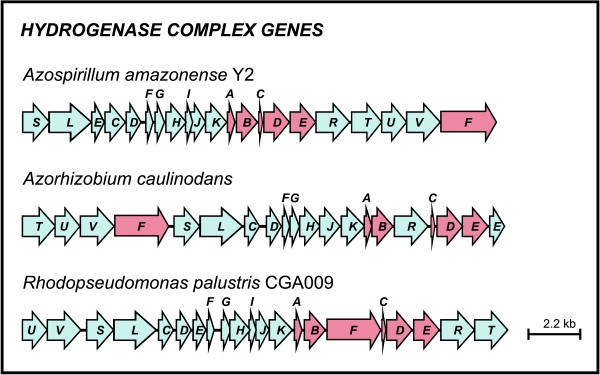
**Organization of the hydrogenase gene cluster across different species from the orders Rhodospirillales and Rhizobiales**. Arrows represent genes and their respective direction of transcription. *hup *genes are colored in red and *hyp *genes are colored in blue.

Nitrogen fixation forms molecular hydrogen (H_2_) as a byproduct. Therefore, diazotrophic bacteria have several hydrogenase systems that are responsible for oxidizing molecular hydrogen to recover part of the energy expended during nitrogenase activity. Genes encoding for an uptake NiFe hydrogenase (*hupSL*) were identified in *A. amazonense *(Figure 6). Furthermore, the accessory proteins required for maturation of the subunits [39,40], encoded by the *hup *and *hyp *genes, are situated downstream of the genes encoding *hupSL (*Figure 6*)*. This organization resembles that found in members of the order Rhizobiales, where, in general, the *hup *and *hyp *genes are clustered (Figure 6), although their ordering is quite heterogeneous among the species. In contrast with this observation, comparisons with closely-related species showed that *Azospirillum *sp. B510 and *A. brasilense *Sp245 have their *hup *and *hyp *genes scattered across the genome. Moreover, the bacteria *R. centenum *does not have the hydrogenase gene cluster.

**Figure 7:**
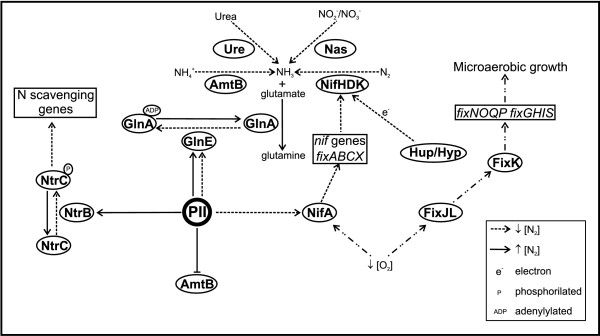
**Integrated model correlating nitrogen metabolism and energy related-pathways for nitrogen fixation**. The legend box indicates the correlation of each symbol with its respective meaning.

An integrated model relating the components discussed in this section with nitrogen metabolism is depicted in Figure 7, taken into consideration the similarity of *A. amazonense *genes to those from other well-known bacterial systems.

### Phytohormone production

Species of the *Azospirillum *genus can positively influence plant growth and crop yield by the biosynthesis and secretion of indole-3-acetic acid (IAA) [2,41]. However, although *A. amazonense *strains are able to synthesize IAA [10], very little is known about the molecular mechanisms responsible for this process.

In *A. brasilense*, at least three pathways for IAA biosynthesis exist, two tryptophan-dependent pathways (indole-3-acetamide pathway (IAM) and indole-3-pyruvate pathway (IPyA)) and one tryptophan-independent pathway [3,42]. Similarly, the genome of *Azospirillum *sp. B510 contains genes responsible for the IAM pathway [7]. However, the *iaaM, iaaH *and *ipdC *genes, related to the IAM or IPyA pathways, were not located in the *A. amazonense *genome. Further analysis of the genome sequence of *A. amazonense *revealed a gene encoding a protein with about 70% similarity to nitrilases from plant species, like *Arabidopsis thaliana *and *Zea mays*, which catalyze the conversion of indole 3-acetonitrile to IAA [43,44]. Future studies may verify if this gene is implicated in IAA biosynthesis in *A. amazonense*.

### Quorum sensing and biofilm formation

Quorum sensing is an intercellular signaling process implicated in the regulation of several traits of bacteria, notably antibiotic biosynthesis and biofilm formation. The archetype for quorum sensing regulation is the LuxIR system, which involves an acyl-homoserine lactone (AHL) synthase (LuxI homolog) and an AHL-dependent transcriptional regulator (LuxR homolog) [45]. The quorum sensing phenomenon of *Azospirillum *species is strain-specific and seems to regulate functions linked to rhizosphere competence and adaptation to plant roots [46].

The acyl-homoserine lactone (AHL) biosynthesis ability of forty *Azospirillum *strains (including *A. amazonense *Y2) was previously investigated, and only four *A. lipoferum *strains seemed of being capable of synthesizing these compounds [47]. However, the genome analysis of *A. amazonense *revealed the presence of genes encoding for LuxI and LuxR homologs proteins (Additional file 1). Therefore, these results indicate that *A. amazonense *Y2 could synthesize AHLs and respond to its presence in the environment. The genome of *A. amazonense *also presents a *Klebsiella pneumoniae ahlK *homolog [48], a predicted gene that codes for a putative homoserine lactonase (Additional file 1) implicated in AHL degradation. Since bacterial plant pathogens rely on quorum sensing mechanisms to infect plants [45], a study of *A. amazonense *homoserine lactonase activity on the deleterious activities of these pathogens would be relevant.

Extracellular polysaccharides are loosely bound to the cell surface and play an important role in bacterium-plant interactions through the firm and irreversible anchoring of cells to the plant roots [3]. In the *A. amazonense *genome, two genes, *noeJ *(mannose-6-phosphate isomerase, Additional file 1) and *noeL *(GDP-mannose 4,6-dehydratase, Additional file 1), which are related to extracellular polysaccharide biosynthesis and biofilm formation, were also found [49].

### Chemotaxis/Motility

Different species of *Azospirillum *attach to and colonize plant root surfaces and these processes depend on chemotaxis. Azospirilla exhibit chemotaxis towards sugars, amino acids, organic acids and root exudates [50]. This ability offers the bacteria the advantage of moving towards favorable nutrient conditions.

Genes encoding for the central signal transduction pathway for chemotaxis (*che*) are present in nearly all motile bacteria. This signal transduction system is composed of the conserved *cheAWYBR *genes and a group of transmembrane chemoreceptors (known as MCPs or methyl-accepting proteins) that perceive environmental signals. Homologs of the *cheAWYBR *and MCP genes were identified in the *A. amazonense *genome (Additional file 1), and some *che *genes display similar organization to those found in the model organism *Escherichia coli *[51].

The *A. amazonense *Che1 gene cluster (*cheAWYBR*) revealed a conserved organization with the major chemotaxis gene cluster from *A. brasilense *Sp245, which modulates cell length and clumping behavior [52]. Apparently, this gene cluster also affects the production of exopolysaccharide and flocculation of *A. brasilense*. Other *che*-like genes (Che2 and Che3, Additional file 1) are present in the *A. amazonense *genome, probably encoding parallel signal transduction pathways that could have distinct functions, similar to those found in *R. centenum *[53,54].

It is also worth noting that genes encoding MCP domain proteins are spread throughout the *A. amazonense *genome. Some of them were classified accordingly to their similarities to *E. coli *chemoreceptors. All five types of *E. coli *MCP receptors were found in the *A. amazonense *genome (Additional file 1), and it is probable that some of them are related to cell motility by regulating the histidine kinase CheA that phosphorylates a response regulator, which in turn controls the rotational direction of the flagellar motor [55].

The flagellum is a key structure for the chemotactic response. In the *A. amazonense *genome, 39 flagellar genes were identified, and the majority of them are distributed among different gene clusters (Additional file 1). The reduced flagellar gene number in *A. amazonense *compared to those of the closely-related *A. brasilense *(79 annotated genes) and *R. centenum *(72 genes) species is in agreement with previous evidence indicating that *A. amazonense *synthesizes only the polar flagellum for swimming motility [56].

**Figure 8:**
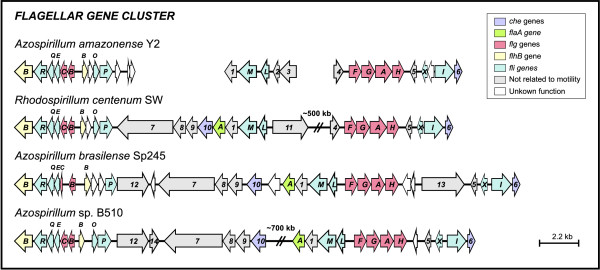
**Organization of the flagellar gene cluster across different species**. Arrows represent the genes and their respective direction of transcription. Genes are colored as depicted in the upper box. 1- ATPase involved in DNA repair, 2- glyoxalase/bleomycin resistance protein/dioxygenase, 3- twin-arginine translocation pathway signal, 4- peptidylprolyl isomerase, 5- RNA polymerase-binding protein DksA, 6- chemotactic signal-response protein CheL, 7- tetratricopeptide TPR_2 (cell wall/envelope/membrane biosynthesis), 8- chemotaxis MotB protein, 9- PPE-repeat protein, 10- chemotaxis protein CheZ, a-flaA, 11- transglutaminase-like domain protein, 12- K+ transporter, 13- O-methyltransferase, 14- beta-Ig-H3/fasciclin.

Homologs of the *A. brasilense *and *R. centenum *genes *fliFHN-motA*-*flbD*-*flhAF-fleN *[9,57] are present in *A. amazonense *(Additional file 1). The *A. amazonense *genome also contains other flagellar genes that display similar organization to those found in *Azospirillum *spp. and *R. centenum *(Figure 8).

Previous studies demonstrated that the transcription factor FlbD is related to the biosynthesis of lateral flagella in *A. brasilense *and the polar flagella in *R. centenum *[9,57]. The presence of the *flbD *gene in the *A. amazonense *genome suggests that it could have a similar regulatory function as that found in *R. centenum*.

### Transport, antibiotics resistance and lantibiotic production

Transport systems allow the uptake of nutrients and ions, excretion of end products of metabolism and communication between cells and the environment.

Several components of the cationic efflux pump, the ATP-Binding Cassette (ABC) transporter superfamily, the Major Facilitator Superfamily (MFS) and the TonB-dependent transporters were identified in the *A. amazonense *genome.

TonB-dependent transport is a mechanism of active uptake across the outer membrane normally related to iron uptake, signal transduction and environmental perception [58,59]. *A. amazonense *has a high number of genes encoding TonB receptors (Additional file 1) when compared with other diazotrophs such as *Azospirillum *sp. B510 (9 annotated genes) and *R. centenum SW *(32 annotated genes). Homology analyses suggest that a set of putative TonB receptors for specific substrates like ferrioxamine, cobalamin (B12 vitamin) and heme are present in this bacterium.

Among the different families of transporters, only two occur ubiquitously in all kingdoms of life: the Major Facilitator Superfamily (MFS) and the ATP-Binding Cassette (ABC) superfamily, representing the largest and most distributed families of transmembrane proteins. MFS proteins are single polypeptide secondary carriers that utilize uniport, symport or antiport mechanisms to transport various small substrates [60]. The ABC transporter proteins utilize energy from adenosine triphosphate (ATP) hydrolysis to carry out the uptake of essential nutrients and/or the extrusion of toxic substances [61]. In the *A. amazonense *genome, several genes encoding for putative MFS and ABC transporters were identified, and they could be implicated in the transport of a wide range of putative substrates (Additional file 1).

Multidrug resistance (MDR) transporters increase drug excretion through an efflux pump, which expels a wide variety of toxic products from the cell, playing a central role in bacterial drug resistance. The MDR transporters belong to various transporter families [62]. In *E. coli*, the transport of diverse substrates out of the cell by the AcrAB-TolC efflux transporter can confer broad resistance to antibiotics [63]. The *acrA *and *acrB *genes normally form an operon whose transcription is regulated by the *acrR *gene product, and are found in the *A. amazonense *genome.

Putative drug resistance transporters of the QacA subfamily were found in the *A. amazonense *genome (Additional file 1), which could confer resistance to monovalent and bivalent cationic lipophilic antiseptics and disinfectants such as quaternary ammonium compounds [64].

Further analysis revealed that, in addition to the MDR transporters, a set of genes whose products could be related to specific antimicrobial resistance are present in the *A. amazonense *genome. Genes that code for penicillin, glyoxalase/bleomycin and tetracycline resistance are also present in the *A. amazonense *genome (Additional file 1). These findings corroborate the experimental data that shows that *A. amazonense *is tolerant to tetracycline and resistant to penicillin [6].

Lantibiotics are peptide-derived antibacterial substances produced by some bacteria, and are characterized by the presence of unusual amino acids like lanthionines and dehydrated amino acids [65]. Lantibiotic biosynthesis is frequently coregulated as part of a stress response when cells enter the late-log or stationary phase [66]. Most lantibiotics exert their antibiotic effect by either forming pores in the target cell membrane or by inhibiting cell wall synthesis, and many lantibiotics are bactericidal against a variety of Gram-positive bacteria [67]. Genes related to lantibiotic synthesis were found in *A. amazonense *genome (Additional file 1).

The genes implicated in antibiotic resistance and in lantibiotic production are probably essential for successful establishment of this microorganism in the soil due to constant contact with niche competitors like fungi and other bacteria.

### Bacteriophytochrome

The *A. amazonense *genome also harbors a bacteriophytochrome gene (Additional file 1). Similarly, *Azospirillum *sp. B510 has two genes that code for distinct types of bacteriophytochromes [7], and *Bradyrhizobium *sp. possesses three bacteriophytochrome genes [68]. In plants, phytochromes regulate the metabolic response to the light environment, but a variety of functions is found in other organisms [69]. The bacteriophytochromes in *R. palustris *regulate the biosynthesis of the photosynthetic apparatus [70], while in *Deinococcus radiodurans *and *R. centenum*, they regulate pigment biosynthesis [71]. Subsequent studies must be carried out to understand the role of the bacteriophytochrome in *A. amazonense *physiology.

## Conclusion

In order to thrive, bacteria must adapt readily to environmental shifts by means of a wide variety of genotypic and phenotypic accommodations [72]. The rhizosphere is a good example of a dynamic environment, where fluctuations in its biological and chemical activities demand an appropriate response from its inhabitants. The species *A. amazonense *is a free-living plant growth-promoting rhizobacterium that is found in association with plants of agricultural importance. In this study, we identified a series of *A. amazonense *genes that could be essential for adaptation to the competitive environment of the rhizosphere. Its wide genetic repertoire confers a versatile metabolism (e.g. the ability to use different carbon and nitrogen sources), as well as different mechanisms of perceiving and exploring its surroundings. These characteristics could directly influence plant growth, for instance, by providing nitrogen and stimulatory compounds to plants. Another important finding was the greater similarity of some genes, e.g. nitrogenase and RubisCO genes, to genes of members of the order Rhizobiales than to genes from other *Azospirillum *species. This evidence illustrates the genetic plasticity of this species and indicates that evolutionary phenomena like horizontal gene transfer could be fundamental for adaptation to its environment.

The major impact of this work will be to guide subsequent studies for a better understanding of the potential of *A. amazonense*.

## Methods

### Bacterial strain, culture conditions, and DNA isolation

*A. amazonense *Y2 (ATCC 35120) was cultured in M79 medium (10 g/L of sucrose as a carbon source, 0.1 g/L of K_2_HPO_4_, 0.4 g/L of KH_2_PO_4_, 0.2 g/L of MgCl_2_.7H_2_O, 0.1 g/L of NaCl, 0.4 g/L of yeast extract, pH 6.5) [73] with shaking at 150 rpm and 35°C for 18 hours. Genomic DNA was isolated as described by Wilson [74]. The quality of the isolated genomic DNA was assessed by agarose gel electrophoresis.

### Genome sequencing, assembly, draft annotation

Total genomic DNA was sequenced using the Roche 454 pyrosequencing platform following the manufacturer's instructions. The contigs were assembled using Newbler software version 2.3 with the default parameters. The estimated coverage of the genome was 35x. Some gaps present in the genes of interest were filled in by sequencing PCR fragments obtained from genomic DNA.

The annotation and analysis of the sequences were carried out using the System for Automated Bacterial Integrated Annotation (SABIA) [75]. This Whole Genome Shotgun project has been deposited at DDBJ/EMBL/GenBank under the accession AFBX00000000. The version described in this paper is the first version, AFBX01000000.

### Phylogenetic analysis

Gene sequences were retrieved from GenBank. Most of the 16S rDNA sequences were retrieved from the Ribosomal Database Project [http://rdp.cme.msu.edu/] [76,77]. The accession numbers of the sequences utilized in the phylogenetic reconstructions are listed in Additional file 4.

Multiple sequence alignments were performed using MUSCLE version 3.8 [78] and CLUSTALW (built into the MEGA 4 software) [79]. Phylogenetic trees were inferred using the neighbor-joining method (1000 bootstrap replicates) by the MEGA 4 software [80]. The evolutionary distances were computed using the Maximum Composite Likelihood method for the nucleotide sequences and the Jones-Taylor-Thornton (JTT) matrix-based method for the amino acid sequences. All positions containing gaps and missing data were eliminated from the datasets (complete deletion option).

## Authors' contributions

ISS and ATRV conceived of and coordinated the study. FHS and RC extracted the genomic DNA. LGPA carried out the draft genome sequencing and assembly. FHS, LGPA, RC, LAR, FMS, MRSM and ISS performed the draft genome annotation. FHS, MRSM and RC carried out the comparative analyses. FHS carried out the phylogenetic analyses and created the illustrations. FHS, RC, LAR, FMS, MRSM and ISS analyzed the results. FMS and LAR wrote some sections of the manuscript. FHS and ISS wrote the manuscript. All authors read and approved the final manuscript.

## Supplementary Material

Additional file 1 **Supplementary table 1**. Genes of *Azospirillum amazonense *described throughout the study. Click here for file

Additional file 2 **Supplementary Figure **1. Domain composition of the NifE and NifU proteins among bacteria from the orders Rhodospirillales and Rhizobiales. The oblong boxes represent protein domains which are colored according to the description in the legend. Click here for file

Additional file 3 **Supplementary table 2**. Putative sigma N and NifA binding sites occurring upstream of some *nif *and *fix *genes. Click here for file

Additional file 4 **Supplementary table 3**. Accession numbers of the sequences utilized in the phylogenetic reconstructions. Click here for file
