# Supplementary material for: Genomic insights into the versatility of the plant growth-promoting bacterium Azospirillum amazonense
Source: BMC Genomics. 2011 Aug 12;12:409. doi: 10.1186/1471-2164-12-409 (PMC3169532; doi:10.1186/1471-2164-12-409)
Supplement: Additional file 2 — Supplementary Figure 1. Domain composition of the NifE and NifU proteins among bacteria from the orders Rhodospirillales and Rhizobiales. The oblong boxes represent protein domains which are colored according to the description in the legend. [file 1471-2164-12-409-S2.PDF]

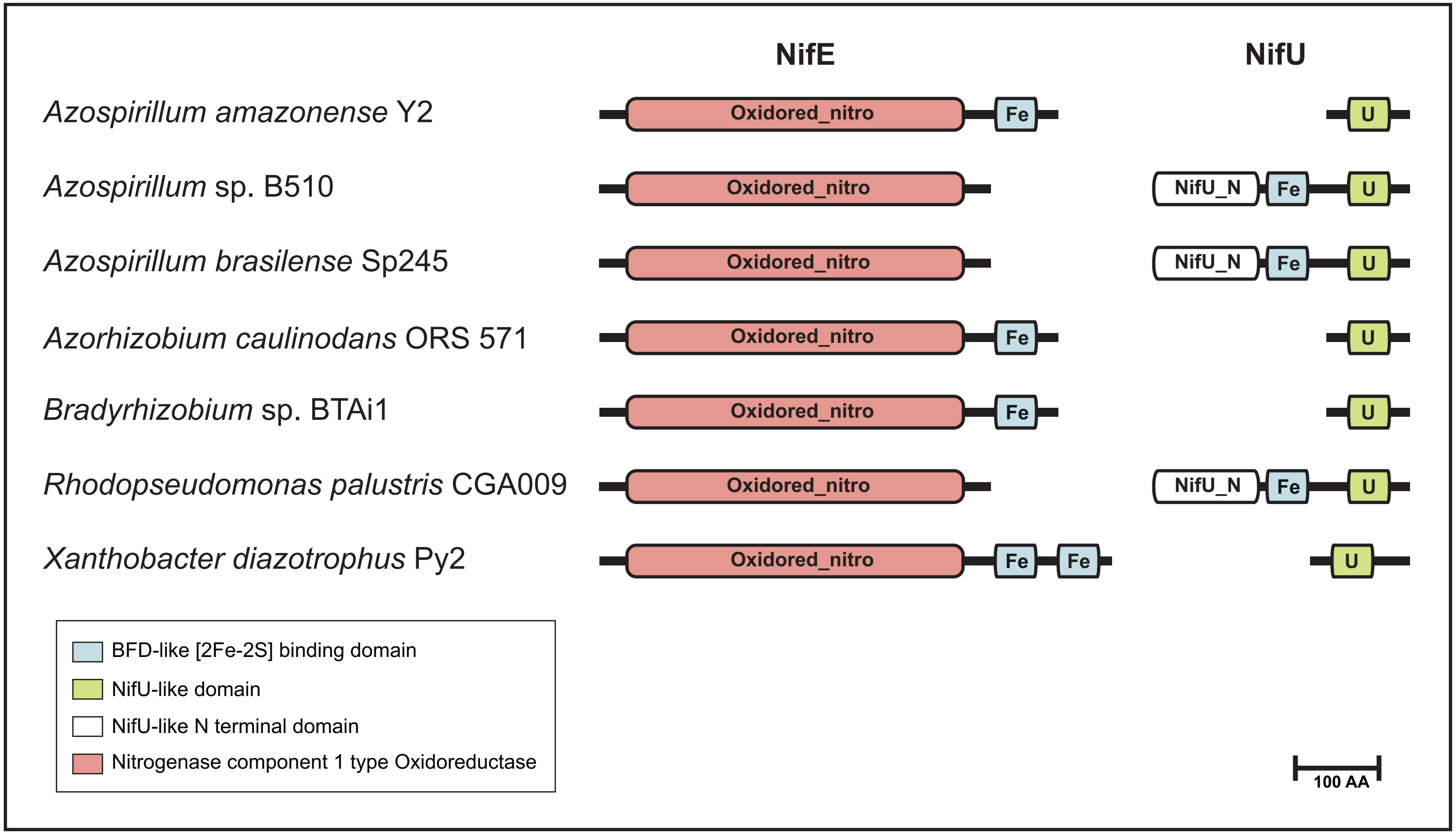

Figure S1 - Domain composition of the NifE and the NifU proteins among bacteria from the orders Rhodospirillales and Rhizobiales. The oblong boxes represent the protein domains which are colored following the description of the legend box.
